# Detection of target DNA using fluorescent cationic polymer and peptide nucleic acid probes on solid support

**DOI:** 10.1186/1472-6750-5-10

**Published:** 2005-04-25

**Authors:** Frédéric R Raymond, Hoang-Anh Ho, Régis Peytavi, Luc Bissonnette, Maurice Boissinot, François J Picard, Mario Leclerc, Michel G Bergeron

**Affiliations:** 1 Centre de recherche en infectiologie de l'Université Laval, Centre hospitalier universitaire de Québec, Pavillon CHUL, Sainte-Foy, Québec, G1V 4G2, Canada; 2 Canada Research Chair in Electroactive and Photoactive Polymers, Département de Chimie, Université Laval, Sainte-Foy, Québec, G1K 7P4, Canada

## Abstract

**Background:**

Nucleic acids detection using microarrays requires labelling of target nucleic acids with fluorophores or other reporter molecules prior to hybridization.

**Results:**

Using surface-bound peptide nucleic acids (PNA) probes and soluble fluorescent cationic polythiophenes, we show a simple and sensitive electrostatic approach to detect and identify unlabelled target nucleic acid on microarray.

**Conclusion:**

This simple methodology opens exciting possibilities for applied genetic analysis for the diagnosis of infections, identification of genetic mutations, and forensic inquiries. This electrostatic strategy could also be used with other nucleic acid detection methods such as electrochemistry, silver staining, metallization, quantum dots, or electrochemical dyes.

## Background

Classical strategies for nucleic acid detection require labelling the analyte or the probes with fluorophores or other reporter molecules [1,2]. Labelling steps add cost, often complexify the reaction mixture, and are not useful when rapidity of the assay is essential, such as for some molecular diagnostic applications. Fluorescent conjugated polymers have already been shown to allow the sensitive detection of DNA and RNA in liquid phase by complexation of the cationic fluorescent polymer to negatively-charged nucleic acids [3-8]. The use of DNA/RNA-binding fluorescent polymers on microarray could allow unlabelled nucleic acid detection, and thus prevent the need for a nucleic acid labelling step. Using PNA capture probes, we show a simple and sensitive electrostatic approach which enables the direct detection and specific identification of unlabelled target nucleic acid analyte using a standard microarray scanner.

## Results and discussion

Experiments with commercially available aldehyde-functionalized glass slides, while giving strong signal when fluorophore-labelled target DNA hybridized to DNA probes, gave no signal when detection was conducted using PNA probes (data not shown). We developed aminoalkylsilane slides activated with carbonyldiimidazole and compared them with commercial aldehyde slides. Our chemistry was compatible with detection on PNA capture probes using Cy3-labelled oligonucleotides or using the fluorescent cationic polymer, while aldehyde slides gave no signal. The aminated slides activated by carbodiimidazole were thus chosen to immobilize both DNA and PNA capture probes for all experiments described here.

**Figure 1:**
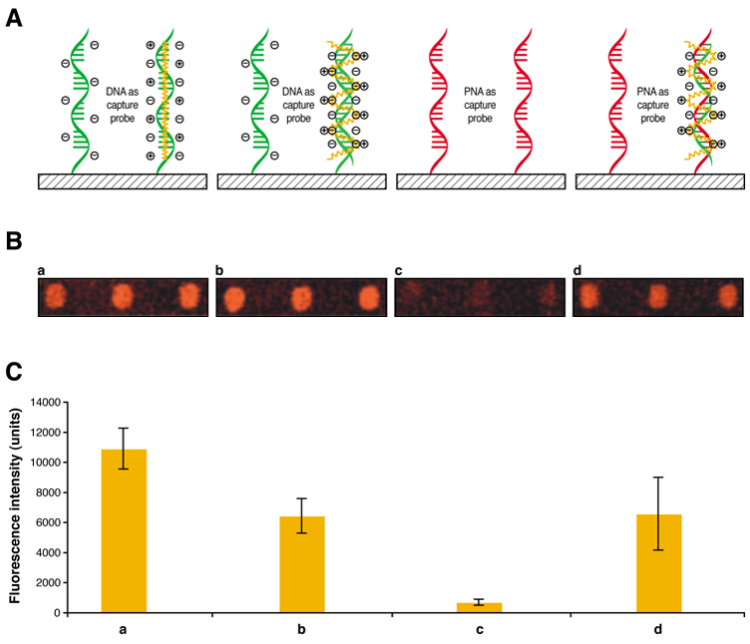
**Cationic polythiophene transducer for the fluorometric detection of hybridization on microarrays. **A) Schematic depiction of the interaction between cationic polymers and a) single-stranded DNA, b) double-stranded DNA, c) single-stranded PNA and d) PNA-DNA duplex. Fluorescent cationic polymer is shown in yellow, DNA probes are shown in green and PNA probes are shown in red. B) Experimental results for fluorometric detection on microarray when cationic polythiophene transducer is reacted with a) single-stranded DNA, b) double-stranded DNA, c) single-stranded PNA and d) PNA-DNA duplex. Results are shown in triplicate. C) Graphs showing the fluorescence intensity with standard deviation for each triplicate shown in B.

Fluorescent conjugated polythiophene derivative used in this study binds nucleic acids via electrostatic interactions with negatively-charged phosphate groups of the DNA backbone [4,5]. This is illustrated in Figure 1 where single-stranded and double-stranded DNA oligonucleotides (Figure 1a and 1b) both produce fluorescent signals due to the formation of DNA-polythiophene complexes. Thus, discrimination between hybridized and non-hybridized DNA capture probes is not possible using a conventional microarray fluorescence scanner.

Capture probes of neutral charge, such as PNA [9-14], have been successfully used in microarray experiments [6,15]. Since PNA do not have a charged backbone, they could be used to allow the detection of nucleic acids with cationic polymeric biosensors such as polythiophenes. Using PNA as capture probes provide a simple and sensitive electrostatic approach on solid support which enables the direct detection and specific identification of unlabelled target nucleic acid analyte with a standard microarray scanner.

The cationic polymer does not bind to unhybridized neutral PNA capture probes (Figure 1c), but strongly interacts with the negatively-charged backbone of the complementary oligonucleotides bound to PNA probes, allowing transduction of hybridization into a fluorescence signal (Figure 1d). This clearly demonstrates the usefulness of PNA capture probes for the detection of hybridization events with positively-charged fluorescent conjugated polymers on solid support. In a recent study, Gaylord *et al*. have shown detection in solution of a complementary DNA hybridized to a PNA probe using Förster resonance energy transfer (FRET) between a water soluble conjugated polymer and a PNA probe labelled with a reporter chromophore [6]. The present study shows similar results without the need for labelled PNA. Moreover, we demonstrate that this detection can be performed using PNA capture probes tethered onto a solid support. Those results suggest that this electrostatic strategy could also be used with other nucleic acid detection methods such as electrochemistry [16], silver staining [17,18], metallization [19,20], quantum dots [21-23], or electrochemical dyes [24].

**Figure 2:**
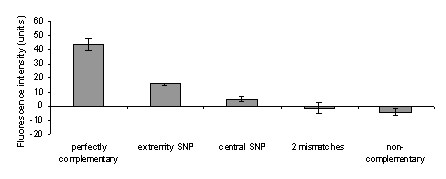
**Specificity of oligodeoxyribonucleotide hybridization to PNA probes. **Hybridizations were performed at room temperature with a concentration of 7.5 × 10^10 ^targets per μL using the fluorescent cationic polymer for detection. Hybridization of PNA probes to perfectly complementary, or complementary oligonucleotides presenting a terminal mismatch, a central mismatch, or two mismatches were performed in triplicate. Fluorescence intensities from hybridized probes were corrected by substraction of background fluorescence intensity.

Specificity of detection was investigated by hybridizing mismatched oligonucleotides to PNA probes. After room temperature hybridization of oligonucleotides with PNA probes, the fluorescent polythiophene polymeric biosensor gave a strong signal over background when target oligonucleotide was fully complementary to the capture probe. Oligonucleotides with two mismatches and non complementary oligonucleotides produced near-background signals easily distinguishable from the much stronger signal observed with perfectly matched hybrids (21-fold stronger) (Figure 2). For a single mismatch, discrimination is related to the position of the mismatch in the capture PNA probe. When the mismatch is located at the probe extremity, signal intensity is reduced 2.5 fold as compared to the perfect match. By contrast, a ratio of 6 is observed when the mismatch is located close to the center of the capture probe (Figure 2).

The sensitivity of the detection scheme described here is approximately 2.5 × 10^-13 ^mole of oligonucleotide in a volume of 20 μL. In a recent report, Nilsson and Inganäs [8] have described the use of a zwitterionic polythiophene derivative able to detect 2 × 10^-8 ^mole of oligonucleotide within a hydrogel matrix. Our approach, based on standard glass slide microarray technologies, is approximately five orders of magnitude more sensitive. Moreover, it is expected that further progress in terms of sensitivity should be obtained by reducing the size of the microarray spots and of the hybridization reaction volume. Also, the detection of larger DNA molecules (e.g. amplicons) should increase sensitivity since the amount of complexed fluorescent cationic polymer is theoretically proportional to the amount of possible electrostatic interactions. The development of scanners specifically fabricated to excite at the wavelength of this fluorescent cationic polymer should also contribute to increase the analytical sensitivity. Indeed, recent optimizations of the fluorometric detection applied to our polymer technology has enabled the detection of only a few hundred molecules of genetic material in 3 mL of aqueous solution [3]. This clearly indicates the great potential of cationic conjugated polymers as highly sensitive fluorescent transducers.

## Conclusion

In this study, we presented a simple and specific nucleic acid detection method onto solid support which does not require labelling of the analyte prior to hybridization. This methodology opens new possibilities for genetic analysis applied for the diagnosis of infections, identification of genetic mutations, and forensic inquiries. For instance, this technology would be useful for the identification of pathogens and related antimicrobial resistance genotypes using microarrays. Finally, the electroactivity of the present fluorescent cationic polymer could be exploited for a real-time electrical discrimination of single nucleotide polymorphisms (SNPs) onto solid support.

## Methods

### Materials

All chemical reagents were obtained from Sigma-Aldrich Co. (St. Louis, MO) and were used without further purification unless otherwise stated. Fluorescent polythiophene used in this study, poly(1H-imidazolium, 1-methyl-3-[2-[(4-methyl-3-thienyl)oxy]ethyl]-, chloride), was prepared following published procedures [3,4]. Oligodeoxyribonucleotide capture probes, which were 5'-modified by the addition of two nine carbon spacers and an amino-linker, were synthesized by Biosearch Technologies (Novato, CA). PNA capture probes having a N-terminal amine and two O linkers were synthesized by Applied Biosystems (Foster City, CA). The amino-linker modification allowed covalent attachment of probes onto a functionalized glass surface. The capture DNA or PNA probe of 15-mer (5'-CCGCTCGCCAGCTCC-3') targeted a polymorphic region of the *bla*_SHV-1 _gene associated with β-lactam antibiotic resistance. Target oligonucleotides (i) fully complementary to the capture DNA or PNA probe (5'-GGAGCTGGCGAGCGG-3'), (ii) having two mismatched bases (5'-GGCGCTGACGAGCGG-3'), (iii) having a central single mismatch (5'-GGAGCTGACGAGCGG-3'), (iv) having a single mismatch at one extremity (5'-GGCGCTGGCGAGCGG-3'), and (v) non complementary (5'-CGCTCTGCTTTGTTATTCGG-3') were synthesized by Biosearch Technologies.

### Preparation of glass slide

The aldehyde-functionalized commercial glass slides were purchased from CEL Associates (Pearland, TX). The home made aminoalkylsilane-functionalized glass slides were prepared as follows. All chemical reactions were carried out in polypropylene jars. Surfaces used were 25 mm × 75 mm microscope glass slides (VWR International, West Chester, PA). After sonication (1 hour) in deionized water, the slides were sonicated in 40 mL of 10% sodium hydroxide for 1 hr, washed several times with deionized water, and dried under a stream of nitrogen. The slides were then sonicated in an aminopropyltrimethoxysilane solution (2 mL water, 38 mL methanol and 2 mL aminopropyltrimethoxysilane) for 1 hr, washed with methanol, dried and baked at 110°C for 15 min. The amine modified slides were activated by sonication overnight in 40 mL of 1.4-dioxane containing 0.32 g (2 mmoles) of carbonyldiimidazole as coupling agent, washed with dioxane and diethyl ether, and dried under a stream of nitrogen.

### Microarray production

The probes were diluted two-fold by the addition of Array-it Microspotting Solution Plus (TeleChem International, Sunnyvale, CA), to a final concentration of 5 μM. Capture probes were spotted in triplicate, using a SDDC-2 arrayer (Bio-Rad Laboratories, Hercules, CA) with SMP3 pins (TeleChem International). Upon spotting, each spot had a volume of 0.6 nL, a diameter between 140 and 150 μm, and contained approximately 1.8 × 10^9 ^amino-modified probes. After spotting, slides were dried overnight, washed by immersion in boiling 0.1% Igepal CA-630 (Sigma-Aldrich, St. Louis, MO) for 5 min, rinsed in ultra-pure water for 2 min, and dried by centrifugation for 5 min under vacuum (SpeedVac plus; Thermo Savant, Milford, MA). Slides were stored at room-temperature in a dry and oxygen-free environment.

### DNA microarray hybridization, polymeric detection and data acquisition

Prehybridization and hybridization were performed in 15 × 13 mm Hybri-well self-sticking hybridization chambers (Sigma-Aldrich). Microarrays were first prehybridized for 15 min at room temperature with 20 μL of 1X hybridization solution (6X SSPE [Omnipur, EM Science, Gilbstown, NJ], 0.03% polyvinylpyrrolidone [PVP], and 30% formamide). Subsequently, the prehybridization buffer was blown out of the chambers and replaced with the same buffer containing the target oligonucleotide (complementary, central mismatch or two mismatches) at a final concentration of 2.5 μM, except for sensitivity experiments for which concentrations ranged from 0.25 nM to 2.5 μM. Hybridization was carried out at 22°C for 15 min. After hybridization, the liquid was expelled from the chambers and replaced by an aqueous solution of cationic polymer (7.3 × 10^-4 ^M based on monomeric units). After a 15 min incubation period, the slides were washed with deionized water containing 0.1 % Igepal CA-630. Microarrays were dried by centrifugation at 1350 × g for 3 min. Slides were scanned using the Cy3 configuration (excitation wavelength at 530 nm) of ScanArray 4000XL (Packard Bioscience Biochip Technologies, Billerica, MA) and the fluorescent signals were analyzed using QuantArray software (Packard Bioscience Biochip Technologies).

## Authors' contributions

FRR participated in the design of the experiments, performed all microarray experiments and co-drafted the manuscript with HAH. HAH synthesized the cationic polythiophene, worked on surface chemistry, and co-drafted the manuscript with FRR. RP supervised some of the work and participated in the design of the experiments. LB conceived the study and participated in the design of the experiments. RP, MB, FJP, ML, and MGB provided guidance and suggestions for experimental design, analyzed data and edited the manuscript. ML holds the Canada Research Chair in Electroactive and Photoactive Polymers. MGB is head of the Centre de recherche en infectiologie de l'Université Laval. All authors read and approved the final version of the manuscript.
